# Supplementary material for: Determinants of substance use among adolescents aged 10–19 years in Tanga Region, Eastern Tanzania: A cross-sectional analysis
Source: PLOS Glob Public Health. 2025 Jul 10;5(7):e0004150. doi: 10.1371/journal.pgph.0004150 (PMC12244608; doi:10.1371/journal.pgph.0004150)
Supplement: Attachment — Submitted filename: Response to Reviewers.docx [file pgph.0004150.s003.docx]

**Point-by-point response to the editorial and reviewer comment**

**Manuscript title:** Determinants of Substance Use Among Adolescents Aged 10-19 Years in Tanga Region, Eastern Tanzania: A Cross-Sectional Analysis.

**Manuscript ID:** PGPH-D-24-02968

Dear editor,

Thank you for the opportunity to revise and resubmit our manuscript titled “Determinants of Substance Use Among Adolescents Aged 10-19 Years in Tanga Region, Eastern Tanzania: A Cross-Sectional Analysis.” (Manuscript ID: PGPH-D-24-02968). We appreciate the constructive feedback provided by you and the reviewers, which has helped us improve our manuscript. Below, we address each of the comments raised.

**Editor Comments:**

**Comment 1**: Please include a complete copy of PLOS’ questionnaire on inclusivity in global research in your revised manuscript.

**Response 1:** Thank you for your comment we have included a complete copy of PLOS’ questionnaire on inclusivity in global research in our revised manuscript.

**Comment 2**: In the online submission form, you indicated that “The data of this study will be given out upon reasonable request to the other”.

All PLOS journals now require all data underlying the findings described in their manuscript to be freely available to other researchers, either
a. In a public repository,
b. Within the manuscript itself, or
c. Uploaded as supplementary information.

**Response 2:** We acknowledge the editor’s comment. The data used in this study was provided by a third-party. We have updated the data availability statement as stated below:

**“**The data analyzed in this manuscript was provided by a third-party source, the Africa Academy for Public Health ([AAPH](https://aaph.or.tz)), in Dar es Salaam, Tanzania. All requests to access the data can directly sent to the Chief Executive Officer of AAPH at [info@aaph.or.tz](mailto:info@aaph.or.tz).”

**Reviewer 1**

**Major Weaknesses and Areas for Improvement:**

**Comment 1:** The study includes 1,262 adolescents, with 1,031 in-school and 231 out-of-school participants. While the sample size is adequate, the relatively small number of out-of-school adolescents (18.3% of the total sample) may limit the generalizability of the findings to this subgroup. The authors should discuss this limitation and consider whether the sample size is sufficient to draw robust conclusions about out-of-school adolescents.

The study was conducted in Tanga City, which may not be representative of other regions in Tanzania or sub-Saharan Africa. The authors should acknowledge this limitation and suggest caution in generalizing the findings to other settings.

**Response 1:** Thank you for your valuable recommendation and constructive feedback, we have added the explanation based on the suggestion. Changes are in the section of Study strengths and limitations, page 18.

**Comment 2:** The study relies on self-reported data, which is prone to recall bias and social desirability bias. While the authors mention efforts to mitigate these biases (e.g., ensuring anonymity, using trained interviewers), they should discuss the potential impact of these biases on the results. For example, underreporting of substance use, particularly among in-school adolescents, could affect the accuracy of the prevalence estimates.

**Response 2:** Thank you for your thoughtful comment. We acknowledge the potential for recall and social desirability bias in self-reported data, we have addressed the comment accordingly Changes are in section of Study strengths and limitations. *“Notwithstanding these strengths the study had some drawbacks.* *Substance use was self-reported, posing the risk of recall and social desirability bias as well as underreporting of substance use”* page 18 line 314-316

**Comment 3:** The cross-sectional design of the study limits the ability to establish causal relationships between determinants and substance use. The authors should explicitly state this limitation and suggest that longitudinal studies are needed to better understand the causal pathways.

**Response 3:** We acknowledge that cross-sectional design limits our ability to establish causal relationships between determinants and substance use. Based on your suggestion, we have explicitly stated this limitation in the manuscript and emphasized the need for longitudinal studies to better understand causal pathways. We appreciate your insight and have incorporated this point into the limitations section. Changes are in section of Study strengths and limitations, page 18 line 319-320 *“the study could not establish a temporal relationship between the exposures and substance use further studies (longitudinal studies) are required for better understand the causal pathways”*

**Comment 4:** The study does not explore psychological or behavioral factors (e.g., mental health, peer influence, family dynamics) that are known to influence substance use.

**Response 4:** We acknowledge that psychological and behavioral factors such as mental health, peer influence, and family dynamics play a role in substance use. However, as noted in the limitations section, these aspects are not typically covered in the Global School-based Student Health Survey (GSHS) and, therefore, could not be analyzed in this study. We have explicitly stated this limitation. Changes are in section of Study strengths and limitations, page 18 Refers manuscript line number (320-321) *“while past research has linked substance use to behavioral and psychological factors, these aspects are not typically covered in the GSHS surveys, hence could not be analyzed in this study”*

**Comment 5:** The authors use modified Poisson regression models to estimate the relative risk (RR) of substance use. While this is appropriate for binary outcomes, the interpretation of the RRs, particularly for current substance use, is challenging due to the small sample size in some subgroups (e.g., only one female reported current substance use). The authors should discuss the potential instability of these estimates and consider alternative statistical approaches, such as logistic regression, which may provide more stable odds ratios (ORs).

**Response 5:** Thank you for your insightful comment. We acknowledge the concern regarding potential instability in the estimates due to the small sample size in some subgroups. Regression analysis was performed using generalized linear models to estimate the determinants of depression and anxiety symptoms. The log-linear regression model utilizes the Poisson family and log-link function, whose relative measure of association is reported as risk ratio. The choice of this model was informed by a rigorous analysis and investigations using the classical logistic regression models, which, as the reviewer mentioned, estimates the odds ratios. However, previous studies (Ref) demonstrated that odds ratios over-estimated prevalence ratios in our analyses. Log-linear models are one of the best alternatives to logistic regression models for the analysis of cross-sectional studies. Furthermore, in our initial analysis, we also experienced non-convergence issues with the use of log-binomial regression models (binomial family with log-link function), which estimate prevalence ratios. We also used Akaike Information Criteria to compare several models, the better fitting model being the log-linear model. “Ref. Barros AJ, Hirakata VN. Alternatives for logistic regression in cross-sectional studies: an empirical comparison of models that directly estimate the prevalence ratio. BMC medical research methodology. 2003 Dec;3:1-3.”

**Comment 6:** The study briefly mentions cultural norms and attitudes toward substance use but does not explore these factors in depth. Given the importance of cultural context in shaping substance use behaviors, the authors should consider incorporating a more detailed discussion of how cultural factors may influence the findings.

**Response 6:** Thank you for your comment. In response, we have expanded the discussion section to provide a more detailed explanation of how cultural norms and societal attitudes may influence substance use behaviors. . Changes are in section of Discussion, page 16, Refers manuscript line number (261-265) *“differences in cultural perceptions of substance use across various communities may contribute to variations in prevalence rates and patterns of use. Future research should explore these cultural dimensions in greater depth, including how societal attitudes, family expectations, and peer influences shape adolescent substance use behaviors”*

**Comment 7:** While the study provides valuable policy recommendations, such as keeping adolescents in school and targeting high-risk groups, the authors could expand on how these interventions might be implemented in practice. For example, what specific strategies could be used to keep adolescents in school? How can digital interventions be effectively leveraged to promote healthy behavior?

**Response 7:** Thank you for the insightful feedback. We have expanded on how the recommended interventions might be implemented in practice, particularly focusing on strategies for keeping adolescents in school and how digital interventions can be effectively utilized to promote healthy behavior. Changes are in section of Conclusion and recommendations, page 19, Refers manuscript line number (328-338)

**Minor Issues**

**Comment 1:** The definitions of "lifetime" and "current" substance use are clear, but the authors should ensure that these terms are consistently used throughout the manuscript. For example, in Table 2, the terms "ever smoked" and "ever drank alcohol" are used, which could be confusing. It would be helpful to align these terms with the definitions provided in the text.

**Response 1:** We appreciate your suggestion to ensure consistency in the use of terms throughout the manuscript. In response, we have aligned the terms "ever smoked" and "ever drank alcohol" with the definitions of "lifetime" substance used in the text to enhance clarity and avoid confusion. Changes are in the section of Results, page 12 Refers manuscript line number (206-207)

**Comment 2:** The results section is well organized, but some tables could be more reader friendly. For example, Table 3 could be simplified by removing redundant information and focusing on the key findings. Additionally, the authors should consider using visual aids (e.g., bar charts or graphs) to illustrate the prevalence of substance use across different subgroups.

**Response 2:** Thank you for your thoughtful feedback. We agree on the importance of making the results section more reader friendly. Given the nature of the data, which includes both "lifetime" and "current" substance use, we opted to present the findings in tables to clearly distinguish between these two groups. Tables allow for structured comparison and enable readers to easily differentiate the prevalence of substance use across the different subgroups, which is especially important for understanding the patterns in both lifetime and current use. The table footnotes have been used to provide detailed information to help the readers understand the information presented in the tables.

**Comment 3:** The discussion section is thorough, but it could benefit from a more critical analysis of the findings. For example, the authors should discuss why older adolescents (15-19 years) are more likely to use substances and how this relates to their greater independence and exposure to risk factors.

**Response 3:** We appreciate your suggestion for a more critical analysis of the findings. In the discussion section, specifically lines 278 to 284, we have already addressed the higher substance use among older adolescents (15-19 years) and provided an explanation for this trend.

**Comment 4:** The conclusion is concise but could be expanded to include a broader discussion of the implications for public health policy and practice. For example, how can the findings be used to inform national or regional strategies to reduce substance use among adolescents?

**Response 4:** We appreciate your comment; we have reshuffled the conclusion respectively. Changes are in section of Conclusion and recommendations, page 19 Refers manuscript line number (328-338) *“Possible approaches to achieve this may involve offering educational assistance, providing vocational training for those who are not in school, and enhancing school engagement through mentoring and counselling initiatives. In addition, targeted behaviors modification programs should focus on high-risk groups such as older adolescents (ages 15-19), youth who are not enrolled in school, males, and individuals who have participated in sexual activities or exhibit other risk factors. Also, the study recommends utilizing digital interventions which provide a valuable means for encouraging healthy habits and informing teenagers about the dangers of substance use. Social media platforms can be utilized to spread educational information, promote positive messages, and increase awareness regarding the impacts of substance use, thereby ensuring extensive reach and involvement with this demographic.”*
