# Supplementary material for: Determinants of substance use among adolescents aged 10–19 years in Tanga Region, Eastern Tanzania: A cross-sectional analysis
Source: PLOS Glob Public Health. 2025 Jul 10;5(7):e0004150. doi: 10.1371/journal.pgph.0004150 (PMC12244608; doi:10.1371/journal.pgph.0004150)
Supplement: Attachment — Submitted filename: Response_to_Reviewers_auresp_2.docx [file pgph.0004150.s004.docx]

**Point-by-point response to the editorial and reviewer comment**

**Manuscript title:** Determinants of Substance Use Among Adolescents Aged 10-19 Years in Tanga Region, Eastern Tanzania: A Cross-Sectional Analysis.

**Manuscript ID:** PGPH-D-24-02968

Dear editor,

Thank you for the opportunity to revise and resubmit our manuscript titled “Determinants of Substance Use Among Adolescents Aged 10-19 Years in Tanga Region, Eastern Tanzania: A Cross-Sectional Analysis.” (Manuscript ID: PGPH-D-24-02968). We appreciate the constructive feedback provided by you and the reviewers, which has helped us improve our manuscript. Below, we address each of the comments raised.

**Reviewer 1**

**Comment 1:** I did not review the original manuscript and will only make a few minor comments. The authors have adequately responded to the points raised in the prior reviews. This manuscript presents finding from data collected on adolescents in the Kanga region of Tanzania. It is nice to see this kind of data being collected and analyzed, and it will, I hope, be part of an ongoing and larger effort to monitor and respond to health risk behaviors in Tanzania and other similar sub-Saharan African countries. The authors made an effort to recruit a sample representative of the in-school population of students in the Kanga region. The recruitment methods for the out-of-school sample are perhaps more vulnerable to selection bias, which is a limitation that should perhaps be highlighted more clearly, since a primary finding of the study is the difference in substance use between the in-school and out-of-school groups. Below are few other points for the authors to consider:

**Response 1:** Thank you for highlighting this important point. We agree that the recruitment approach for the out-of-school sample may introduce some risk of selection bias, especially given the central focus on comparing substance use between in-school and out-of-school adolescents. We have now addressed this limitation more explicitly in the manuscript under the Study strengths and limitations section (lines 329–333), where we note:

"Although the out-of-school sample was recruited through community-based methods, which may carry some risk of selection bias, efforts were made to reach diverse participants. This potential limitation should be considered when interpreting differences in substance use, though the findings remain valuable for highlighting disparities between groups."

We appreciate your suggestion, which has strengthened the transparency of our discussion.

**Comment 2:** It would be helpful for readers to be given some brief information on the Kanga province and how it is similar to or different from other parts of Tanzania and other parts sub-Saharan African countries. Is that region unusually prosperous or poor? Is the economy more or less dependent on agriculture or trade? Are there population-wide measures of public health that would help the reader understand how the Kanga province compares to other parts of sub-Saharan Africa?

**Response 2:** Thank you for your valuable recommendation and constructive feedback, we have added the explanation based on the suggestion. Changes are in the section of Study design and setting, page 6 line 115-133. *“We performed a secondary analysis of cross-sectional survey data of adolescents aged 10 to 19 years, conducted in 2021 in Tanga City, located in the Tanga region of Northern Tanzania, by the Africa Academy for Public Health (AAPH). The primary aim of the original project was to establish a foundation for consistently collecting adolescent health data across various domains to inform efforts aimed at improving global adolescent health and well-being. Tanga City, the administrative and commercial hub of the region, lies along the Indian Ocean in the northeastern part of Tanzania Mainland. It is situated approximately 354 kilometers north of Dar es Salaam and about 250 kilometers south of Mombasa, Kenya. The city borders Muheza District to the west and south, Mkinga District to the north, and the Indian Ocean to the east. With an estimated population of 305,585 and an annual growth rate of 1.2%, adolescents aged 10 to 19 years make up about a quarter of the population. The city is ethnically diverse, home to groups such as the Sambaa, Zigua, Bondei, and Digo each comprising less than 20% of the population while over 40% belong to other ethnic backgrounds. The majority religion is Islam. The population comprises office workers (17%), shopkeepers (20%), industrial workers (17%), and unemployed individuals (46%). Administratively, Tanga City is divided into 4 divisions, 27 wards, and 181 streets. The city council oversees 108 primary schools (80 government and 28 private) and 44 secondary schools (26 government and 18 private), serving a total of 26,202 students. (13)”*

**Comment 3:** The tables could format differently to make them more concise and easier to read. In Tables 1, 2, and 3, binary variables the Ns and %s could be reported for one category since the Ns and %s for the other categories are implied. This would reduce the number of rows. In Table 3, it might not be necessary to provide separate rows and RRs of 1.00 for the reference category.

**Response 3:** Thank you for the constructive feedback regarding the formatting of Tables 1, 2, and 3. We appreciate your suggestion to improve clarity and conciseness by reporting Ns and percentages for only one category of binary variables, and by omitting redundant rows for reference categories in Table 3.

We have revised Table 1 accordingly and agree that the new format enhances readability and reduces unnecessary detail. However, after applying similar formatting changes to Tables 2 and 3, we found that the revised versions were more difficult to interpret and less intuitive, particularly for readers who are less familiar with the dataset or statistical methods. To preserve clarity and ensure the tables remain easy to follow, we have retained the original format for Tables 2 and 3, which we believe supports better understanding of the results.

We hope this explanation is satisfactory and are happy to make further adjustments if needed.

**Comment 4:** The authors used a stepwise regression approach to choose what variables to include in their final models. For this stage in the research, that might be fine (and the authors do present the information for bi-variate associations), but as the authors move forward in their program of research, I would recommend they move toward making model-specification decisions based more on theory or prior research.

**Response 4:** Thank you for the constructive suggestion regarding model specification. While we did use stepwise regression in this study, the selection of variables was also informed by insights from the literature to ensure relevance and alignment with existing research. However, we agree that grounding model specification more explicitly in theory and prior studies is a stronger approach. We will take this recommendation into consideration and aim to apply it more rigorously in future stages of our research program.

**Comment 5:** I suspect that the way the authors coded age may have resulted in not adequately adjusting for age as a confounder when assessing the unique effect of being out-of-school. Age is a strong correlate of alcohol and tobacco initiation. The fairly wide age bins that were used here may not be ideal. Being out of school is likely to be strongly associated with age. It might be nice to present that information (i.e., the average ages of the in-school and out-of-school groups). There is a concern that the out-of-school group may be made up of adolescents aged 18 or 19; the age 15–19 category might not adequately capture that.

**Response 5:** Thank you for this important observation regarding the coding of age and its potential impact on confounding, particularly in relation to school status and substance use. We acknowledge the limitation of using wide age categories, which may have masked important variations and correlations, especially given the strong relationship between age, school status, and behaviors such as alcohol and tobacco initiation.

To address this concern, we have recoded age as a continuous variable in the regression models to more accurately adjust for its potential confounding effect. These changes have now been implemented, and the updated results are reflected in Table 3.

Additionally, for other categorical variables, we assessed potential associations using pairwise correlations (pwcorr) and cross-tabulations in Stata. The observed p-values indicated that many of these associations were statistically significant, supporting the relevance of including these variables in the analysis.
